# Damage-induced neuronal endopeptidase (DINE) enhances axonal regeneration potential of retinal ganglion cells after optic nerve injury

**DOI:** 10.1038/cddis.2017.212

**Published:** 2017-06-01

**Authors:** Aoi Kaneko, Sumiko Kiryu-Seo, Sakiko Matsumoto, Hiroshi Kiyama

**Affiliations:** 1 Department of Functional Anatomy and Neuroscience, Graduate School of Medicine, Nagoya University, 65 Tsurumai-cho, Showa-ku, Nagoya 466-8550, Japan

## Abstract

Damage-induced neuronal endopeptidase (DINE)/endothelin-converting enzyme-like 1 (ECEL1) is a membrane-bound metalloprotease that we identified as a nerve regeneration-associated molecule. The expression of DINE is upregulated in response to nerve injury in both the peripheral and central nervous systems, while its transcription is regulated by the activating transcription factor 3 (ATF3), a potent hub-transcription factor for nerve regeneration. Despite its unique hallmark of injury-induced upregulation, the physiological relevance of DINE in injured neurons has been unclear. In this study, we have demonstrated that the expression of DINE is upregulated in injured retinal ganglion cells (RGCs) in a coordinated manner with that of ATF3 after optic nerve injury, whereas DINE and ATF3 are not observed in any normal retinal cells. Recently, we have generated a mature DINE-deficient (KO^Tg^) mouse, in which exogenous DINE is overexpressed specifically in embryonic motor neurons to avoid aberrant arborization of motor nerves and lethality after birth that occurs in the conventional DINE KO mouse. The DINE KO^Tg^ mice did not show any difference in retinal structure and the projection to brain from that of wild–type (wild type) mice under normal conditions. However, injured RGCs of DINE KO^Tg^ mice failed to regenerate even after the zymosan treatment, which is a well-known regeneration-promoting reagent. Furthermore, a DINE KO^Tg^ mouse crossed with a *Atf3*:BAC Tg mouse, in which green fluorescent protein (GFP) is visualized specifically in injured RGCs and optic nerves, has verified that DINE deficiency leads to regeneration failure. These findings suggest that injury-induced DINE is a crucial endopeptidase for injured RGCs to promote axonal regeneration after optic nerve injury. Thus, a DINE-mediated proteolytic mechanism would provide us with a new therapeutic strategy for nerve regeneration.

Regeneration of damaged axons is crucial for successful functional repair in the adult central nervous system (CNS). The regenerative capacity of adult mammalian CNS neurons is limited compared with that of peripheral nervous system (PNS) neurons.^1^ In line with this, retinal ganglion cells (RGCs) hardly regenerate after optic nerve injury. Multiple positive and negative regulators, whose manipulation enhances the intrinsic regenerative ability, have been reported.^1, 2^ For instance, the forced expression of cytokines, such as ciliary neurotrophic factor and leukemia inhibitory factor, and the macrophage-activating factors, such as the zymosan, induces axonal regeneration after optic nerve injury.^3, 4, 5^ Deletion or co-deletion of the phosphatase and tensin homologue and the suppressor of cytokine signaling 3 in injured RGCs induces robust optic nerve regeneration.^6, 7^ However, more precise mechanisms underlying optic nerve regeneration remain to be elucidated.

Damage-induced neuronal endopeptidase (DINE) is a neuronal membrane-bound metalloprotease, which we identified as a nerve regeneration-associated molecule and termed from its unique property;^8^ the expression of DINE is markedly upregulated in injured neurons following both PNS and CNS injuries. The transcriptional regulation of DINE in response to nerve injury is partly regulated by cytokines such as leukemia inhibitory factor and its co-receptor gp130-mediated signaling pathway.^8, 9^ In addition, the transcriptional response of DINE to nerve injury is coincident with that of activating transcription factor 3 (ATF3), which is a stress-responsive transcription factor known as an axonal injury marker.^10, 11^ Our promoter analysis of *Dine* has demonstrated that injury-inducible transcription factors, such as ATF3, STAT3 and cJun, form a complex together with Sp1 that is a general transcription factor after nerve injury.^12^ Concomitantly, recent large-scale bioinformatics analyses have revealed that the coordinated response of core transcription factors, such as ATF3, cJun and STAT3, induce regeneration-associated genes (RAGs).^13^ In other transcriptome analyses using control and injured retinal tissues, ATF3 and DINE are presented within the 10 most upregulated genes.^14, 15^ Based on these results, it is likely that injury-induced DINE is involved in promoting axonal regeneration or determining the fate of injured neurons. However, the physiological relevance of DINE in injured neurons has not yet been elucidated.

The functional significance of DINE *in vivo* has been demonstrated by gene manipulation strategies. The conventional DINE knockout (KO) mouse, which was initially generated by us and others, dies immediately after birth because of respiratory failure,^16, 17^ which is caused by aberrant arborization of motor nerves in target muscles and impaired neuromuscular junction formation in muscles including respiratory muscles. DINE belongs to the M13 protease family, which includes amyloid *β* degrading enzyme, neprilysin, and endothelin-converting enzyme.^18^ Although these family members share a high sequence similarity around the protease active domain, only DINE has not been shown to have any obvious proteolytic activity *in vitro* against multiple candidate substrates such as amyloid *β*, big-endothelin, galanin, and somatostatin.^19, 20^ It is possible that the *in vitro* system is not sufficient for DINE to activate as a protease for some unknown reasons, because an *in vivo* rescue study crossing DINE KO mice with a couple of mutant DINE-expressing transgenic mice has revealed that the enzymatically active domain of DINE is essential^21^; therefore, DINE could be a physiologically crucial enzyme in the nervous system. Apart from our group, several independent groups have reported that endothelin-converting enzyme-like 1 (ECEL1; the human homologue of DINE) is a responsible gene for type 5 distal arthrogryposis (DA) in human, which is a congenital contracture disorder.^22, 23^ Subsequently, we have found that aberrant arborization of motor axons and failure of neuromuscular formation can be a primary cause of DA with the ECEL1 mutation.^24^ In line with this, it is likely that DINE has an important role as a protease in the neurons where its expression is abundant.

In the above-mentioned study, we have succeeded in producing mature DINE-deficient (KO^Tg^) mouse by crossing with the DINE overexpressing transgenic mouse in embryonic motor neurons, which avoids lethality and demonstrates normal development after birth.^21^ Using the mouse, in this study we have clarified that DINE-ablated RGCs fail to regenerate even after treatment with the regeneration-promoting reagent zymosan. Furthermore, crossing of the mature DINE KO^Tg^ mouse with an injury-inducible GFP (*Atf3*:BAC Tg) mouse in which exogenous GFP is induced under the control of *Atf3* regulatory sequences,^25^ verified that DINE deficiency impairs regenerative competence. These data suggest that injury-induced DINE is crucial to enhance intrinsic regenerative ability to promote axonal regeneration, probably through the proteolytic activity.

## Results

### The expression of DINE is increased in injured RGCs

**Figure 1:**
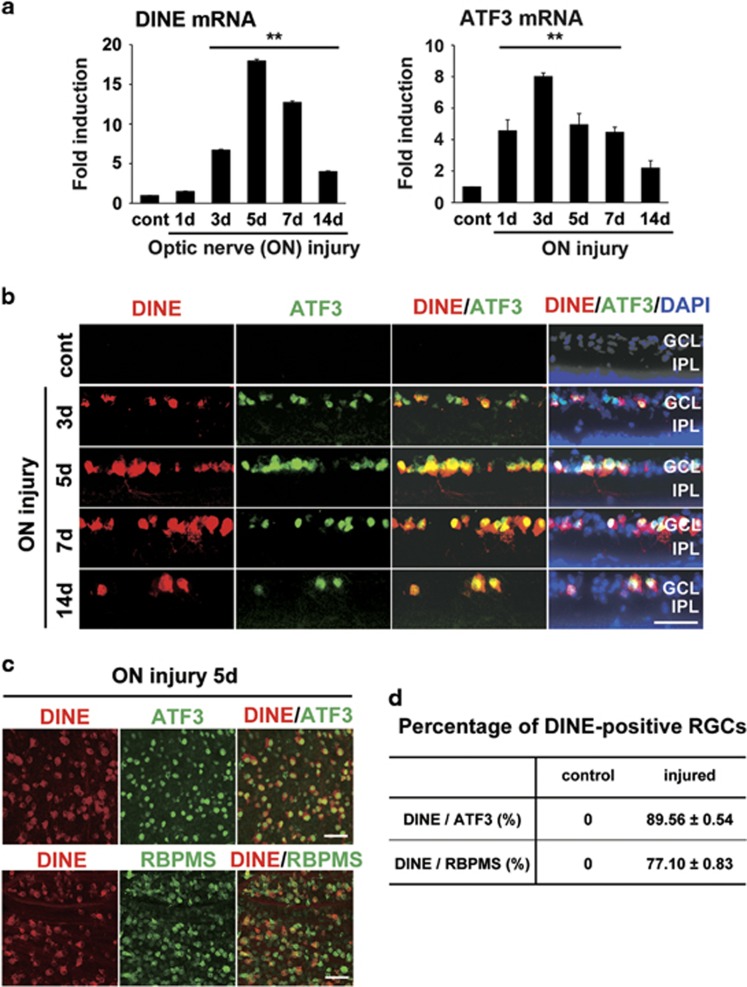
The expression of damage-induced neuronal endopeptidase (DINE) in injured retinal ganglion cells (RGCs) after optic nerve injury. (**a**) Quantitative RT-PCR of DINE and activating transcription factor 3 (ATF3) mRNAs in retinal tissues at 1 day (1d), 3 days (3d), 5 days (5d), 7 days (7d), and 14 days (14d) after optic nerve crush injury. GAPDH expression served as a reference. Data are shown as a fold induction compared with mRNA expression in control retina. *n*=3 mice at each point, **P<0.001, according to one-way ANOVA followed by Tukey’s *post-hoc* test. (**b**) Immunohistochemistry of retinal sections using anti-DINE (red) and anti-ATF3 (green) antibodies and DAPI (blue) after optic nerve injury. (**c**) Representative images of whole-mount preparation of retinae at 5 days after optic nerve injury. The immunoreactivities using anti-DINE (red), anti-ATF3 (green) and anti-RNA-binding protein with multiple splicing (RBPMS; green) antibodies were observed in RGCs. (**d**) DINE, RBPMS- or ATF3-positive RGCs were counted in whole-mount retina prepared in (**c**) and presented as the percentage of DINE-positive cells in RBPMS- or ATF3-positive RGCs. Data are the mean±S.E.M. *n*=4 mice per group. cont, control; GCL, ganglion cell layer; IPL, inner plexiform layer. Scale bars, 50 *μ*m in (**b**) and (**c**)

We firstly examined expression of DINE mRNA in retinal tissues after optic nerve crush. Quantitative reverse-transcription PCR (qRT-PCR) showed that the expression of DINE mRNA was upregulated at 3 days, reached a peak level at 5 days and was then downregulated at 14 days after injury (Figure 1a). Compared with that of ATF3, the mRNA induction profile of DINE was slightly delayed. Immunohistochemical staining using an anti-DINE antibody further revealed that the protein expression of DINE was exclusively detected in injured RGCs in a similar time-dependent manner as observed with mRNA expression (Figure 1b). The immunoreactivity of DINE in injured RGCs was also well colocalized with that of ATF3. To further characterize DINE-positive RGCs, we performed whole-mount immunohistochemistry of the injured retina using anti-DINE, anti-ATF3 and anti-RNA-binding protein with multiple splicing (RBPMS) antibodies, and counted the number of positive RGCs for each (Figures 1c and d). Because RBPMS is a selective marker of pan-RGCs before and after injury, this was used for identification of RGCs.^26^ DINE-positive cells occupied approximately 90% of ATF3-positive cells and 80% of RBPMS-positive cells, suggesting that DINE is induced in a majority of injured RGCs after optic nerve injury.

### DINE KO (KO^Tg^) mouse shows similar retinal structure with WT mouse

**Figure 2:**
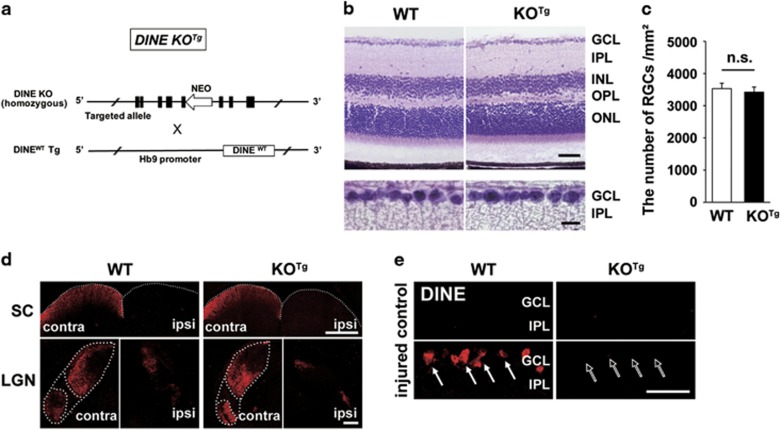
The retinal structure in wild-type (WT) and DINE knockout (DINE KO^Tg^) mice. (**a**) Schematic illustration of DINE KO^Tg^ mouse generation. In DINE KO^Tg^ mice, the *Dine* genes of both alleles are targeted. The exogenous DINE^WT^ is expressed transiently in embryonic motor neurons, because DINE^WT^ transgene is regulated under the control of embryonic motor neuron-specific Hb9 promoter. Black box indicates exon. (**b**) Retinal sections of WT and DINE KO^Tg^ mice stained with hematoxylin and eosin. Lower panels show magnified image of ganglion cell layer (GCL) in upper panels. (**c**) The number of RGCs in WT and DINE KO^Tg^ mice. Whole-mount immunohistochemistry of intact retina was performed using an anti-RBPMS antibody. Data are presented as the number of RBPMS-positive RGCs per area (mm^2^) and the mean±S.E.M. *n*=4 mice per group. n.s., not significant, according to Student’s *t*-test. (**d**) Innervation of optic nerves to the superior colliculus (SC) and the lateral geniculate nucleus (LGN). LGN is surrounded by dotted lines. Cholera toxin B-positive axons are similarly shown in the target areas in WT and DINE KO^Tg^ mice. (**e**) Expression of DINE protein in the retinal sections at 5 days after optic nerve injury (arrows). DINE-deficient RGCs never showed DINE immunoreactivity even after optic nerve injury (outlined white arrows). IPL, inner plexiform layer; INL, inner nuclear layer; OPL, outer plexiform layer; ONL, outer nuclear layer; contra, contralateral; ipsi, ipsilateral. Scale bars, 50 *μ*m in (**b**, upper panel) and (**e**), 10 *μ*m in (**b**, lower panel), 500 *μ*m in (**d**, upper panel), and 200 *μ*m in (**d**, lower panel)

To address an effect of DINE deficiency in injured RGCs, we generated DINE KO^Tg^ mice. The DINE KO^Tg^ mouse had homozygous target alleles of *Dine* genes, which deleted DINE conventionally, and carried the transgene, whose expression was regulated under the control of an Hb9 promoter, to express WT DINE specifically in embryonic motor neurons (Figure 2a). Under normal conditions, hematoxylin–eosin staining did not show any difference in the retinal layer or RGCs structure between WT and KO^Tg^ mice (Figure 2b). To further confirm this, we counted the number of RGCs using whole-mount immunohistochemistry with the anti-RBPMS antibody in normal retina from WT and KO^Tg^ mice (Figure 2c). There was no significant difference in RGC number between WT and KO^Tg^ mice. Next, we injected the anterograde tracer cholera toxin B (CTB) into the vitreous body to observe the axonal projection of the optic nerve to the targeted nuclei, the superior colliculus (SC) and the lateral geniculate nucleus (LGN) under normal conditions. The CTB-positive area in the SC and LGN was similar in WT and KO^Tg^ mice (Figure 2d), suggesting that the optic nerves of DINE KO^Tg^ mice project to target regions normally during development. Overall, we concluded that the retinal structure of the DINE KO^Tg^ mouse was comparable to that of the WT mouse. To further assess the injury-induced response, we examined the expression of DINE in RGCs of WT and DINE KO^Tg^ mice before and after optic nerve crush injury (Figure 2e). In normal retina, there was no positive signal of DINE in either WT or DINE KO^Tg^ mice. After optic nerve injury, marked expression of DINE was observed in RGCs of WT mice, whereas the expression was absent in those of DINE KO^Tg^ mice.

### Number of RGCs after nerve injury is not altered in DINE KO^Tg^ mice

**Figure 3:**
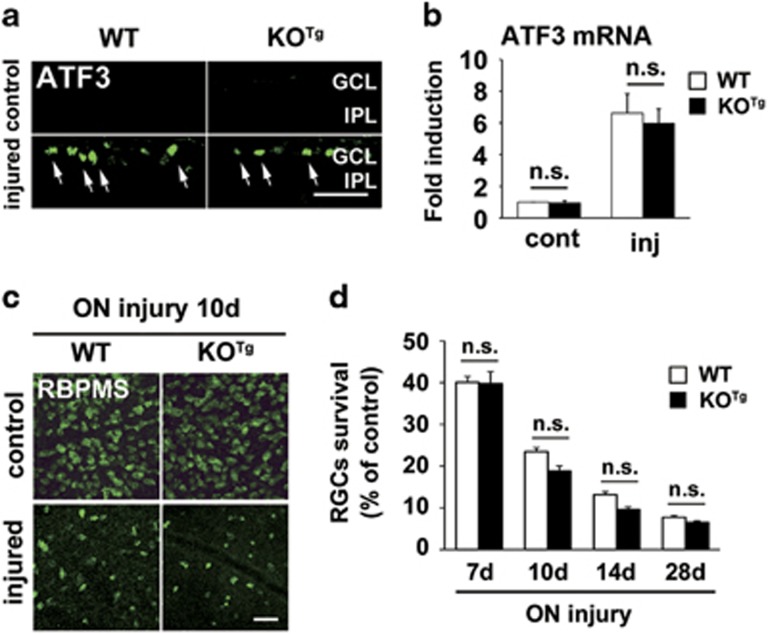
DINE deficiency does not affect the survival of injured RGCs severely. (**a**) Immunohistochemical staining for ATF3 in retinal sections of WT and DINE KO^Tg^ mice at 5 days after optic nerve injury. Arrows indicate ATF3-positive RGCs. (**b**) qRT-PCR of ATF3 mRNA in the retina of WT and DINE KO^Tg^ mice at 5 days after optic nerve injury. GAPDH expression served as a reference. Data are shown as a fold induction compared with mRNA expression in control retina cont, control; inj, injured. *n*=3 mice per group. The significance was determined by ANOVA followed by Tukey’s *post-hoc* test. n.s., not significant. (**c**) Representative images of whole-mount preparation of retinae at 10 days after optic nerve injury. Surviving RGCs were visualized by immunostaining using an anti-RBPMS antibody. (**d**) Quantification of surviving RGCs in retinal whole-mounts of WT and DINE KO^Tg^ mice at 7 days (7d), 10 days (10d), 14 days (14d) and 28 days (28d) after optic nerve injury. RBPMS-positive cells of control and injured retinae were counted. Data are presented as the percentage of surviving injured RGCs compared with control RGCs and the mean±S.E.M. *n*=7–10 mice. The significance was determined by ANOVA followed by Tukey’s *post-hoc* test. n.s., not significant; GCL, ganglion cell layer; IPL, inner plexiform layer. Scale bars, 50 *μ*m in (**a** and **c**)

Next, we compared the expression level of ATF3 following optic nerve injury in WT and KO^Tg^ mice to examine whether the induction of ATF3 is influenced by DINE deficiency. qRT-PCR and immunohistochemistry demonstrated that the expression of ATF3 in both mice after optic nerve injury was comparable (Figures 3a and b), suggesting that absence of DINE does not affect expression of ATF3, which is also crucial in transcriptional regulation of other regeneration-associated molecules. To explore the resistance against injury, we performed whole-mount immunohistochemistry of the retina using an anti-RBPMS antibody and counted the number of RBPMS-positive surviving RGCs (Figures 3c and d). The number of surviving RGCs after optic nerve injury was similar between WT and KO^Tg^ mice, although the number in DINE KO^Tg^ mice was slightly reduced. These results suggest that DINE deficiency is not a primary factor for the survival of injured RGCs.

### DINE-deficient RGCs reduce axonal regeneration

We attempted to evaluate the competence of axonal regeneration in WT and KO^Tg^ mice.

**Figure 4:**
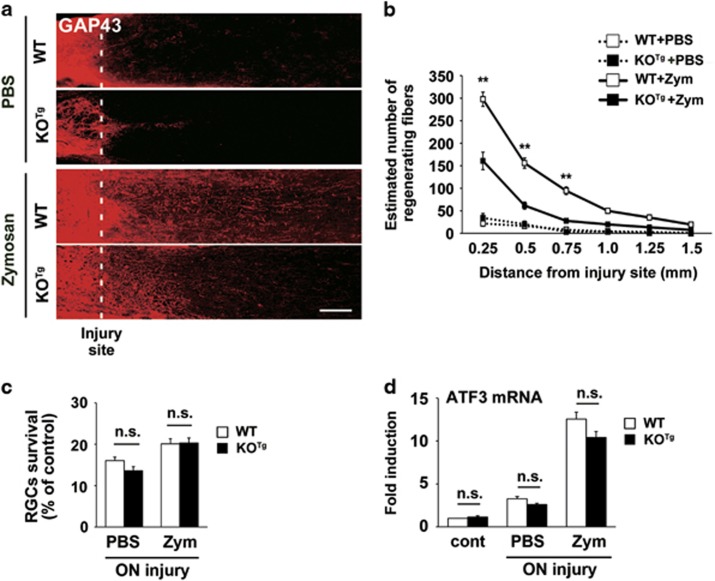
DINE KO^Tg^ mouse significantly reduces axonal regeneration after optic nerve injury coupled with intraocular injection of zymosan. (**a**) Longitudinal sections of optic nerve from WT and DINE KO^Tg^ mice at 14 days after optic nerve crush and intravitreal vehicle (phosphate buffered saline, PBS) or zymosan injection. Regenerating axons were visualized by immunostaining using an anti-GAP43 antibody. The injury site is shown as a dotted line. (**b**) Quantification of the number of GAP43-positive axons at 0.25–1.5 mm from the injury site at 14 days after optic nerve injury. Results are presented as the mean±S.E.M. *n*=5–12 mice per group. **P<0.001, one-way ANOVA followed by Tukey’s *post-hoc* test. Zym, zymosan. (**c**) Quantification of surviving RGCs in retinal whole-mounts of WT and DINE KO^Tg^ mice at 14 days after optic nerve injury and intravitreal vehicle (PBS) or zymosan injection. RBPMS-positive cells of PBS-injected and zymosan-injected retinae were counted. Data are presented as the percentage of surviving injured RGCs compared with control RGCs and the mean±S.E.M. *n*=4–7 mice per group. Zym, zymosan; n.s., not significant according to ANOVA followed by Tukey’s *post-hoc* test. (**d**) qRT-PCR of ATF3 mRNAs in the retinae of WT and DINE KO^Tg^ mice at 14 days after optic nerve injury with intraocular PBS or zymosan (Zym) application. GAPDH expression served as a reference. Data are shown as a fold induction compared with mRNA expression in control retina (cont). n.s., not significant according to ANOVA followed by Tukey’s *post-hoc* test. Scale bar, 100 *μ*m in (**a**)

We applied yeast cell wall extract, zymosan, into the vitreous body on injury, which is a potent monocyte activator and promotes axonal regeneration.^27^ We visualized regenerating fibers with immunohistochemistry using an anti-GAP43 antibody, because GAP43 is a classical marker for axonal regeneration.^27^ At 14 days after optic nerve crush, the zymosan-treated WT mice showed robust axonal regeneration beyond the injury site. In contrast, in the zymosan-treated DINE KO^Tg^ mice, the fibers extended beyond the injury site to significantly lesser extent (Figure 4a). When we quantified the number of GAP43-positive axons at 0.25 to 1.5 mm from injury site, the number of regenerating axons in zymosan-treated WT mice was significantly higher than that in the KO^Tg^ mice (Figure 4b). Control treatment with phosphate-buffered saline (PBS) did not induce obvious fiber regeneration in either WT or KO^Tg^ mice.

We further examined whether regeneration failure of DINE KO^Tg^ mice was caused by the different survival rate of injured RGCs in response to zymosan. Whole-mount immunohistochemistry of retinae using an anti-RBPMS antibody showed that the zymosan treatment after optic nerve injury increased RGC survival rate in both WT and KO^Tg^ mice, compared with PBS application. There was no difference in the number of surviving injured RGCs with or without zymosan between WT and KO^Tg^ mice (Figure 4c). We further investigated the expression of ATF3 in response to zymosan, because ATF3 represents the regenerative potential of injured RGCs. ATF3 mRNA was upregulated in both WT and KO^Tg^ mice at 14 days after optic nerve injury coupled with PBS treatment, compared with that of control (Figure 4d). Injured RGCs with zymosan treatment further upregulated the expression of ATF3 mRNA compared with PBS treatment in both WT and KO^Tg^ mice (Figure 4d). However, the expression level of ATF3 mRNA was slightly lower in KO^Tg^ mice, probably due to degeneration of DINE-deficient RGCs. These data suggest that the DINE-deficient injured RGCs fail to extend axons in response to zymosan even though they have a regenerative potential.

### DINE deficiency in injured RGCs decreases GFP-labeled regenerating axons

**Figure 5:**
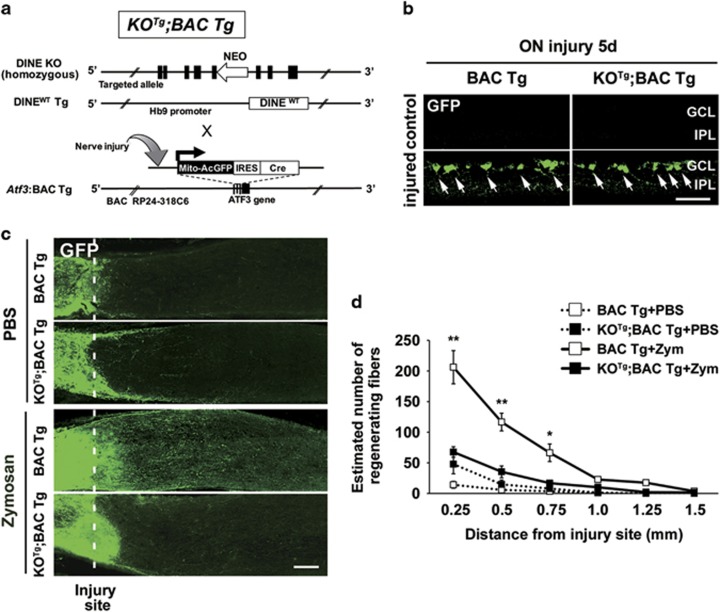
DINE KO^Tg^ mouse fails to extend green fluorescent protein (GFP)-labeled regenerating fibers. (**a**) Schematic illustration of KO^Tg^;BAC Tg mouse generation. DINE KO mice crossed with DINE^WT^ Tg mice were further crossed with injury-inducible GFP mice (*Atf3*:BAC Tg). Black box indicates exon. (**b**) The immunohistochemistry of retinal sections from *Atf3*:BAC Tg (BAC Tg) and KO^Tg^;BAC Tg mice using anti-GFP (green) antibody at 5 days after optic nerve injury. Exogenous GFP labels injured RGCs (arrows). (**c**) Longitudinal sections of optic nerve from BAC Tg and KO^Tg^;BAC Tg mice at 14 days after optic nerve injury coupled with PBS or zymosan treatment. Regenerating axons were visualized by immunostaining using an anti-GFP antibody. The injury site is shown as a dotted line. (**d**) Quantification of the number of GFP-positive signals at 0.25–1.5 mm from crush site at 14 days after optic nerve injury. Zym, zymosan. Results are presented as the mean±S.E.M. *n*=5–6 mice per group. *P<0.05, **P<0.001, one-way ANOVA with Tukey’s *post-hoc* test. GCL, ganglion cell layer; IPL, inner plexiform layer. Scale bars, 50 *μ*m in (**b**) and 100 *μ*m in (**c**)

Recently, we have established the injury-inducible *Atf3*:BAC Tg mouse, which is a bacterial artificial chromosome (BAC) transgenic mouse, using an approximately 200 kb transgene containing the *Atf3* whole genome.^25^ The *Atf3*:BAC Tg mouse expresses GFP specifically in injured neurons, which mimics transcriptional regulation of *Atf*3. Because the mitochondrial targeting signal is attached to GFP in the mouse, the GFP is imported into mitochondria immediately after synthesis, resides in the soma, and is transported into axonal tips and dendrites. To achieve injury-induced GFP-labeling of regenerating axons, we crossed the *Atf3*:BAC Tg mouse with WT or DINE KO^Tg^ mice (Figure 5a). Both BAC Tg mice and KO^Tg^;BAC Tg mice showed a similar response to optic nerve injury and induced the expression of GFP in injured RGCs (Figure 5b). DINE KO^Tg^ mice crossed with *Atf3*:BAC Tg mice lost GFP-labeled regenerating fibers after optic nerve injury, even when coupled with the application of zymosan (Figures 5c and d), further confirming the loss of regenerative capacity in DINE KO^Tg^ mice.

## Discussion

In this study, we have revealed a physiological role of DINE in RGCs after optic nerve injury. We have assumed that DINE could be associated with nerve regeneration because DINE was initially identified in transcriptomic analysis to identify RAGs,^8^ and because the transcriptional response of DINE to nerve injury is regulated by ATF3, which is a core transcriptional factor to initiate nerve regeneration.^9, 10, 12, 13, 28^ Using newly generated DINE KO^Tg^ mice and injury-responsive GFP mice, here we have demonstrated that injury-induced DINE plays a role in axonal regeneration after CNS nerve injury.

After optic nerve injury, the expression of DINE was upregulated in injured RGCs, in which ATF3 was also induced with a slight proceeding timing. The finding supports our previous report that ATF3 is a key transcription factor to induce the expression of DINE following nerve injury.^12, 28^ The present study further confirms that DINE is located at the downstream target of ATF3 after optic nerve injury *in vivo*, because the ablation of DINE in injured RGCs did not alter the expression of ATF3. Conversely, ATF3-deficient RGCs abolished an increase of DINE mRNA after optic nerve injury.^29^ A recent *in silico* study identified ATF3 as one of the hub-transcription factors that activates the intrinsic regeneration program to drive rapid axon elongation in a PNS injury model where injured neurons can regenerate.^13^ Additionally, previous studies have demonstrated that overexpression of ATF3 promotes neurite elongation and neuronal survival.^10, 30^ Therefore, it is feasible that DINE is involved in the gene network system for regeneration downstream of ATF3 after optic nerve injury.

A question arises here regarding why DINE is induced in injured RGCs in conjunction with ATF3, despite the fact that injured RGCs have limited ability in axonal regeneration. One possible explanation may be that multiple core-transcription factors to activate regeneration programming, including the Krüppel-like family of transcription factors, STAT, and Fos, are missing in injured RGCs,^13^ although ATF3-mediated regeneration programming is partly initiated after injury. Therefore, the gene networks including DINE downstream of ATF3 cannot be coordinated with other gene networks to execute nerve regeneration programming. Another possibility is that the expression level and duration may be insufficient to induce or maintain the subsequent responses. Both DINE and ATF3 are transiently upregulated following optic nerve injury, and are downregulated at 14 days after injury. In contrast, PNS neurons, which survive and regenerate after injury, prolong those expressions after nerve injury.^10, 31^ Zymosan treatment of injured RGCs elevated and prolonged the expression of ATF3 in WT mice, thereby leading to robust axonal growth, which was not observed in DINE-deficient RGCs. Taken together, the enhanced and prolonged expression of ATF3 may be necessary to initiate regeneration programming and DINE-mediated mechanism at the downstream of ATF3 to increase the regenerative potential of injured RGCs.

Using DINE KO^Tg^ mice, the present study has demonstrated that injury-induced DINE is critical for injured RGCs to promote nerve regeneration. RGCs are divided to multiple subpopulations.^32^ Among them, osteopontin (OPN)-positive *α*RGCs are considered to have higher regeneration potential. DINE expresses in both osteopontin (OPN)-positive *α*RGCs and OPN-negative other RGCs after optic nerve injury (data not shown). Therefore, DINE seems to have an impact on *α*RGCs with axon-growth potential as well as on other types of RGCs such as M1 RGCs after nerve injury.^33, 34^ These injured RGCs activate and orchestrate multiple signaling pathways, such as mTOR-, STAT3- and Rho-mediated pathways, to enhance robust nerve regeneration^1^.^33, 35^ DINE might be involved in these intracellular signaling pathways. The pathways are activated by extracellular signals, such as growth factors, cytokines and extracellular matrix, which are cleaved by well-characterized metalloproteases such as a disintegrin, metalloproteinase family members and matrix metalloprotease family members.^33, 36, 37^ Intriguingly, previous studies report that these metalloproteases participate in nerve regeneration.^38, 39, 40^ Although DINE is still an orphan protease, our previous study has verified that DINE indeed acts as a protease *in vivo*.^21^ Considering that DINE belongs to the M13 family, injury-induced DINE could modulate the intrinsic state of injured RGCs by activating or inhibiting the signaling pathway through the cleavage of peptides, which might be secreted by activated macrophages or injured RGCs. Furthermore, our previous finding has shown that DINE in embryonic motor neurons has an effect on immature Schwann cells in muscles, which are essential to promote axonal branching and neuromuscular junction formation.^21^ If regenerating RGCs use similar programs as those in developing neurons, it is likely that DINE cleaves some unidentified substrates, presumably peptides, on regenerating axons or the cell body to elicit intercellular signaling between injured neurons and surrounding glial cells or neighboring neurons. Further study will be needed to elucidate the more detailed mechanism of DINE, including substrates during nerve regeneration.

In this study, we additionally used a new tool, the *Atf3*:BAC Tg mouse, which has great potential for studying nerve regeneration.^25^ Using the *Atf3*:BAC Tg mouse, injured axons are readily visualized by GFP without special manipulations such as additional intraocular injection of tracer and histochemical staining. In the present study, the number of GFP-positive regenerating fibers was slightly lower than that of GAP43-imunostained fibers. This could possibly result from an underestimation of GFP-positive regenerating fibers in the *Atf3*:BAC Tg mouse; in the mouse, axons in the space between mitochondria are not counted because GFP is localized on mitochondria, which are visualized as a dot-like structure in the axon. However, the advantages of the mouse exceed this minor limitation. The *Atf3*:BAC Tg mouse could facilitate the study of axonal transport of mitochondria, which is closely associated with axonal growth and axonal degeneration.^25, 41, 42, 43, 44, 45^ Another advantage is that the mice express *Cre* recombinase in response to nerve injury. Although we did not use this *Cre* system in this study, further gene deletion specifically in nerve-injured neurons is possible. Taken together, the use of this mouse model will expand our understanding of the functions of RAGs, including DINE, in nerve regeneration.

In conclusion, we have shown that DINE is a potent peptidase that increases the intrinsic ability of injured RGCs to regenerate after optic nerve injury via a yet unknown mechanism. Exploring the DINE-mediated regeneration pathway, including its substrates, would provide insight into novel therapeutic targets to recover from traumatic injury and to prevent progress of neurodegenerative disorders.

## Materials and Methods

### Animals

DINE KO mice, which we generated previously, were maintained as heterozygous mice on a C57BL/6 background, because homozygous DINE KO mice die after birth.^16^ To obtain mature DINE KO mice, we crossed a heterozygous DINE KO mouse with a DINE^WT^ Tg mouse, which expresses WT DINE in embryonic motor neurons under the control of an Hb9 promoter. Heterozygous DINE KO mice carrying the DINE^WT^ transgene were then crossed with heterozygous DINE KO mice to generate homozygous DINE KO mouse carrying the DINE^WT^ transgene. The resulting DINE-deficient (DINE KO^Tg^) mice can survive and grow to adulthood.^21^ To visualize injured RGCs and axons with GFP, we crossed the DINE KO^Tg^ mouse with an *Atf3*:BAC Tg mouse, which we generated previously.^25^ Tail lysates from these mice were used for genotyping with specific primer sets (Table 1).

All animal protocols were performed in accordance with the University Animal Committee Guidelines for the care and use of laboratory animals and were approved by the Nagoya University Institutional Animal Care and Use Committee. All possible efforts were made to minimize suffering.

### Optic nerve injury and intraocular injection

Optic nerve injury was performed as described previously.^6^ Briefly, animals were anesthetized with inhalation of isoflurane and the right eyelid was incised. A minute incision was made in the sclera and the extraocular muscle was slit along the optic nerve. Then, the nerve was crushed 1 mm behind the eyeball with angled jeweler’s forceps (Dumont #5) for 5 s. Non-operated eyes were used as controls. To promote axon regeneration, zymosan (12.5 *μ*g/*μ*l in PBS, 2 *μ*l) (Sigma, St.Louis, MO, USA) was injected intraocularly at the superior part of the eyeball with a glass needle without damaging the lens on the same day as optic nerve crush. Zymosan was sterilized in boiled water before use. As a control, we used 2 *μ*l of vehicle of PBS.

### RNA preparation and qRT-PCR

Total RNA was extracted from the retinae of three mice using RNeasy Lipid Tissue Mini Kit (Qiagen, Hilden, Germany). Total RNA was converted to cDNA with Superscript Reverse Transcriptase III (Invitrogen, Carlsbad, CA, USA) and nucleotide oligo-dT. Quantitative PCR was performed on a StepOnePlus unit (Applied Biosystems, Waltham, MA, USA) using SYBR Green PCR Master Mix (Applied Biosystems). Primer sets are shown in Table 1.

### Immunohistochemistry

For immunohistochemistry, mice were transcardially perfused with 2% paraformaldehyde (PFA) in PB containing 0.2% picric acid at the appropriate endpoints. Eyes were removed from connective tissues, post-fixed in 2% PFA in PB overnight at 4 °C, and then transferred to 30% sucrose solution. For immunostaining using an anti-DINE antibody, mice were decapitated and the eyes were freshly frozen in dry ice. Both perfused and fresh frozen sections were embedded in OCT compound, cut into serial 16-*μ*m-thick sections on a cryostat, and thaw-mounted onto silane-coated glass slides. Fresh frozen sections were fixed in 4% PFA for 10 min before immunohistochemical procedures. Both sections were washed in 0.01 M PBS, blocked with 1% BSA/0.3% triton X-100 for 30 min, washed in 0.01 M PBS, and incubated with the primary antibodies against goat anti-DINE (Santa Cruz Biotechnology, Dallas, TX, USA), rabbit anti-ATF3 (Santa Cruz Biotechnology), rabbit anti-GAP43 (Millipore, Billerica, MA, USA) and rabbit anti-GFP (MBL, Nagoya, Japan) antibodies at a 1:1000 dilution. After rinsing in 0.01 M PBS, sections were incubated with Alexa Flour 488 or 594-conjugated secondary antibodies (1:500, Invitrogen) at room temperature for 2 h. Following another wash, the sections were mounted and visualized by a fluorescent microscope using a × 40 objective (BZ9000, Keyence, Osaka, Japan).

### Hematoxylin–eosin staining

To observe the retinal structure of WT mice and DINE KO^Tg^ mice, retinal sections were stained with hematoxylin–eosin. The sections were imaged with a fluorescent microscope using × 40 and × 100 objectives (BZ9000, Keyence).

### Optic nerve projection

To evaluate the projection of optic nerves in WT mice and KO^Tg^ mice, 1.5 *μ*l of CTB conjugated to Alexa 594 (1 *μ*g/*μ*l, Invitrogen) was injected into the vitreous chamber using a glass needle. Five to seven days after injection, mice were perfused with 2% PFA in PB containing 0.2% picric acid and the dissected brains were post-fixed in 2% PFA solution overnight at 4 °C. Brains were dehydrated, embedded in OCT compound and cut into 20-*μ*m-thick coronal sections. The SC and LGN were observed with a fluorescent microscope using a × 10 objective (FV10i, Olympus, Tokyo, Japan).

### Whole-mount immunohistochemistry

Mice were transcardially perfused with 2% PFA in PB containing 0.2% picric acid or decapitated. The eyes were removed and fixed in 2% or 4% PFA for at least 3 h. The eyecups were dissected and then incubated in 30% sucrose overnight at 4 °C. The sclera and pigment epithelium were removed, retinae were washed in 0.01 M PBS, incubated in blocking buffer containing 5% goat serum/0.3% triton X-100/PBS overnight at 4 °C and reacted with rabbit anti-RBPMS (1:1000, GeneTex, Irvine, CA, USA) antibody for 5 days at 4 °C. For immunostaining using goat anti-DINE and rabbit anti-ATF3 antibodies, retinae were incubated in blocking buffer containing 5% BSA/0.3% triton X-100/PBS and reacted with primary antibodies for 3 days at 4 °C. After washing in 0.01 M PBS, retinae were incubated with Alexa Flour 488- or 594-conjugated secondary antibodies (1:500, Invitrogen) overnight at 4 °C. Retinae were rinsed in 0.01 M PBS three times and mounted on coverslips.

### Cell count

After whole-mount immunohistochemistry of the retinae, images were acquired in the four quadrants of the retina with a fluorescent microscope using a × 20 objective (BZ9000). The number of immune-positive cells was counted using a 0.25 mm × 0.25 mm grid at 0.5, 1.5, 2.5 mm from the optic nerve head in each quadrant. For cell survival, the number of RBPMS-positive RGCs in each retina was counted and calculated as an average density per mm^2^. To evaluate the proportion of DINE-containing RGCs, RBPMS-, ATF3- and DINE-positive cells were counted.

### Axon regeneration

For analysis of axon regeneration, GAP43- and GFP-positive axons were imaged with a fluorescent microscope using a × 20 objective (BZ9000, Keyence). The number of axons was counted at distances from 0.25 mm to 1.5 mm from the injury site at 0.25 mm intervals. To calculate the number of axons per millimeter of optic nerve, the width of the nerve at the point where axon counting was performed was determined using ImageJ software (NIH, Bethesda, MD, USA). The number of axons per millimeter was averaged across 3–5 sections (GAP43) and 2–4 sections (GFP). The total number of regenerating axons was estimated with this formula: Σ_*d*_=Π*r*^2^ × [average axons/mm]/*t*. In this formula, Σ_*d*_ is the total number of axons at distance *d* from the injury site, *r* is the radius of optic nerve, and *t* is the thickness of the optic nerve sections.

### Statistical analysis

Data were presented as the mean±S.E.M. For statistical comparisons, we used one-way analysis of variance with Tukey’s *post-hoc* test. Significance levels and the number of animals used in each experiment were presented in the figure legends.

## Figures and Tables

**Table 1:** Specific primers used for this study

**Gene**	**Sequence (5’ – 3’)**	**Method**
DINE KO-F	CGCATCGCCTTCTATCGCCTTCTTGACGAG	Genotyping
DINE KO-R1	GCTGGGGGACAGGTGGGAGCTGATGA	Genotyping
DINE KO-R2	CCCCACCAGCCCCGGTTATGTTATCC	Genotyping
DINE^WT^ Tg-F	GATGCCCAGAAGGTACCCCATTG	Genotyping
DINE^WT^ Tg-R	CTTGTACAGCTCATCCATGCC	Genotyping
*Atf3*:BAC Tg-F	CAATAAGATGGAGTACAACTACAACGC	Genotyping
*Atf3*:BAC Tg-R	GACTCTTTCCACAACTATCCAACTCAC	Genotyping
DINE-F	GTCTCTGAACTACGGGGGTATTGGCAC	qRT-PCR
DINE-R	GTAGGCCAGCTTGAGGCCTCCCATGTC	qRT-PCR
ATF3-F	GACTCTTTCCACAACTATCCAACTCAC	qRT-PCR
ATF3-R	TTGACGGTAACTGACTCCAGC	qRT-PCR
GAPDH-F	GGTGAAGGTCGGTGTGAACG	qRT-PCR
GAPDH-R	CGTGAGTGGAGTCATACTGGA	qRT-PCR
